# Plant microphenotype: from innovative imaging to computational analysis

**DOI:** 10.1111/pbi.14244

**Published:** 2024-01-13

**Authors:** Ying Zhang, Shenghao Gu, Jianjun Du, Guanmin Huang, Jiawei Shi, Xianju Lu, Jinglu Wang, Wanneng Yang, Xinyu Guo, Chunjiang Zhao

**Affiliations:** ^1^ Beijing Key Lab of Digital Plant, Information Technology Research Center Beijing Academy of Agriculture and Forestry Sciences Beijing China; ^2^ National Key Laboratory of Crop Genetic Improvement, National Center of Plant Gene Research, Hubei Hongshan Laboratory Huazhong Agricultural University Wuhan China

**Keywords:** computational phenotyping, genetic effects, imaging technique, microphenotype, trait identification

## Abstract

The microphenotype plays a key role in bridging the gap between the genotype and the complex macro phenotype. In this article, we review the advances in data acquisition and the intelligent analysis of plant microphenotyping and present applications of microphenotyping in plant science over the past two decades. We then point out several challenges in this field and suggest that cross‐scale image acquisition strategies, powerful artificial intelligence algorithms, advanced genetic analysis, and computational phenotyping need to be established and performed to better understand interactions among genotype, environment, and management. Microphenotyping has entered the era of Microphenotyping 3.0 and will largely advance functional genomics and plant science.

## Introduction

Water and carbon serve as the basis for all life, and their efficient transport in the soil–plant‐atmosphere (SPC) continuum is essential to sustaining plant growth, development, and reproduction. Water is absorbed by roots from soil, transported through the xylem, and transpired to the atmosphere through stomata in leaves (Mencuccini *et al*., 2019). Carbon is fixed by photosynthetic leaves, transported through the phloem, and stored for storage reserves and growth (Savage *et al*., 2016). Phenotyping traits related to water and carbon processes and understanding their function are of great importance for basic plant science and plant breeding to ensure food security under global climate change. Some of these processes, such as water and sugar transport, have been well described (Jensen *et al*., 2016); however, their operation is poorly understood, as it is specific to cells and tissue. For instance, the structure, organization, and biochemical composition of cells and tissues are essential to shape the structure and function of the vascular system in plants (Strock *et al*., 2022). Although great progress in phenotyping traits at macro levels (organ, plant, and canopy) has been achieved over the past two decades (Tracy *et al*., 2021; Yang *et al*., 2020; Zhao *et al*., 2018), much less attention has been given to phenotyping traits at micro levels and their correlation to those at macro levels (Großkinsky *et al*., 2015). Phenotyping these traits at micro levels will help the precise characterization, accurate identification, and systematic understanding of favourable complex traits (e.g. resource use efficiency) at the macro level.

**Figure 1:**
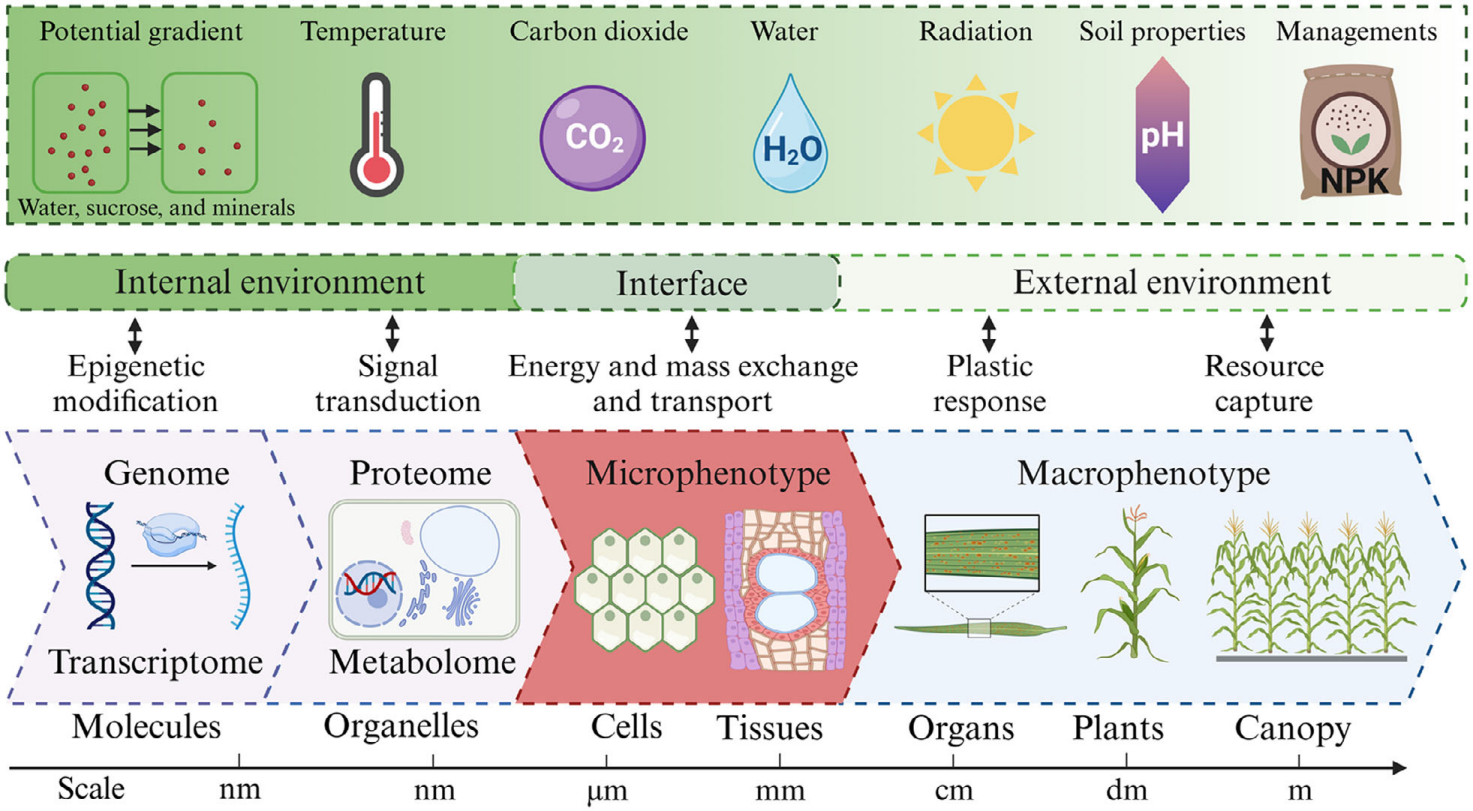
Schematic illustration of microphenotypes, which describes its important role in bridging the genome, transcriptome, proteome, metabolome, and macrophenotypes. Microphenotype is a key interface between metabolome and macrophenotype where plants process environmental cues in the intersection of internal and external environment (Xu *et al*., 2022) mainly through energy and mass exchange between plants and the soil/atmosphere and their transport in plants. Microphenotypes could be used to determine complex macrophenotypes such as water use efficiency (WUE) and radiation use efficiency (RUE) with modelling approaches.

Analogous to the definition of phenotype by Zavafer *et al*. (2023), the set of observable traits at the tissue and cell scales in plants, which could be physical, chemical, or biological, expressed by a genotype at a given time is termed the ‘microphenotype’. Plants capture environmental stimuli at various levels ranging from the canopy to organ and show plastic responses. These stimuli from the external environment are transported through energy and mass exchange and transport at the level of cells and tissues and converted to those in the internal environment. The effects of such internal cues are brought and amplified by means of signal transduction at the level of organelles (Lincoln and Eduardo, 2006). At the molecular level, environmental changes influence gene expression by epigenetic modifications (Li *et al*., 2021). Therefore, the microphenotype plays a key role in bridging the gap between the genotype and complex macrophenotype (Figure 1).

From an agricultural perspective, biophysical and biochemical processes underlying water and carbon cycling, such as photosynthesis, mass flow, partitioning, and their response to environmental factors, are closely related to breeding target traits towards high‐yield, high‐quality, resilient, and resource‐efficient crops. These favourable traits are mainly determined by the sum of physiological processes that are expressed in microphenotypes, necessitating the upscaling of the effects of these microphenotypes to the individual and canopy levels (Roitsch *et al*., 2022; Salon *et al*., 2017; Zhang, Wang, *et al*., 2021). However, these processes mainly occur at the cell and tissue levels and cannot be investigated through macroscopic phenotyping methods (Clark *et al*., 2020; Hall *et al*., 2016). To better understand these physiological processes and provide their implications for favourable traits, innovative microphenotyping modalities ranging from the subcell to suborgan scale and corresponding computational analysis are thus needed.

This review aims to provide an overview of microphenotyping from innovative imaging to computational analysis, with a particular focus on the water and carbon processes in plants. First, we examine the development in microphenotyping in plants over the past two decades. Then, we detail advances in data acquisition and the intelligent analysis of plant microphenotypes from instruments to algorithms. We further evaluate the applications of microphenotyping in basic plant science and plant breeding. In addition, we point out several challenges in microphenotyping studies in crops and suggest that microphenotyping is entering a new stage of development, necessitating novel imaging strategies, extraction algorithms, statistical approaches, and computational phenotyping.

## Progress of microphenotyping research in plants

We analysed the current state, progress, and challenges of research on ‘plant microphenotypes’ in agriculture and plant science by reviewing published literature sourced from the Web of Science database (Clarivate Analytics) using the keywords ‘microphenotype’ and ‘anatomical phenotype’, as well as imaging techniques such as ‘positron emission tomography’ and ‘magnetic resonance imaging’. The search was further narrowed down to include only publications in the fields of agriculture and plant science, resulting in a data set of 12 395 publications. From this data set, we manually selected 513 publications based on their title, abstract, keywords, and methodology and categorized them according to the year of publication, imaging techniques, spatial scale, and organs.

**Figure 2:**
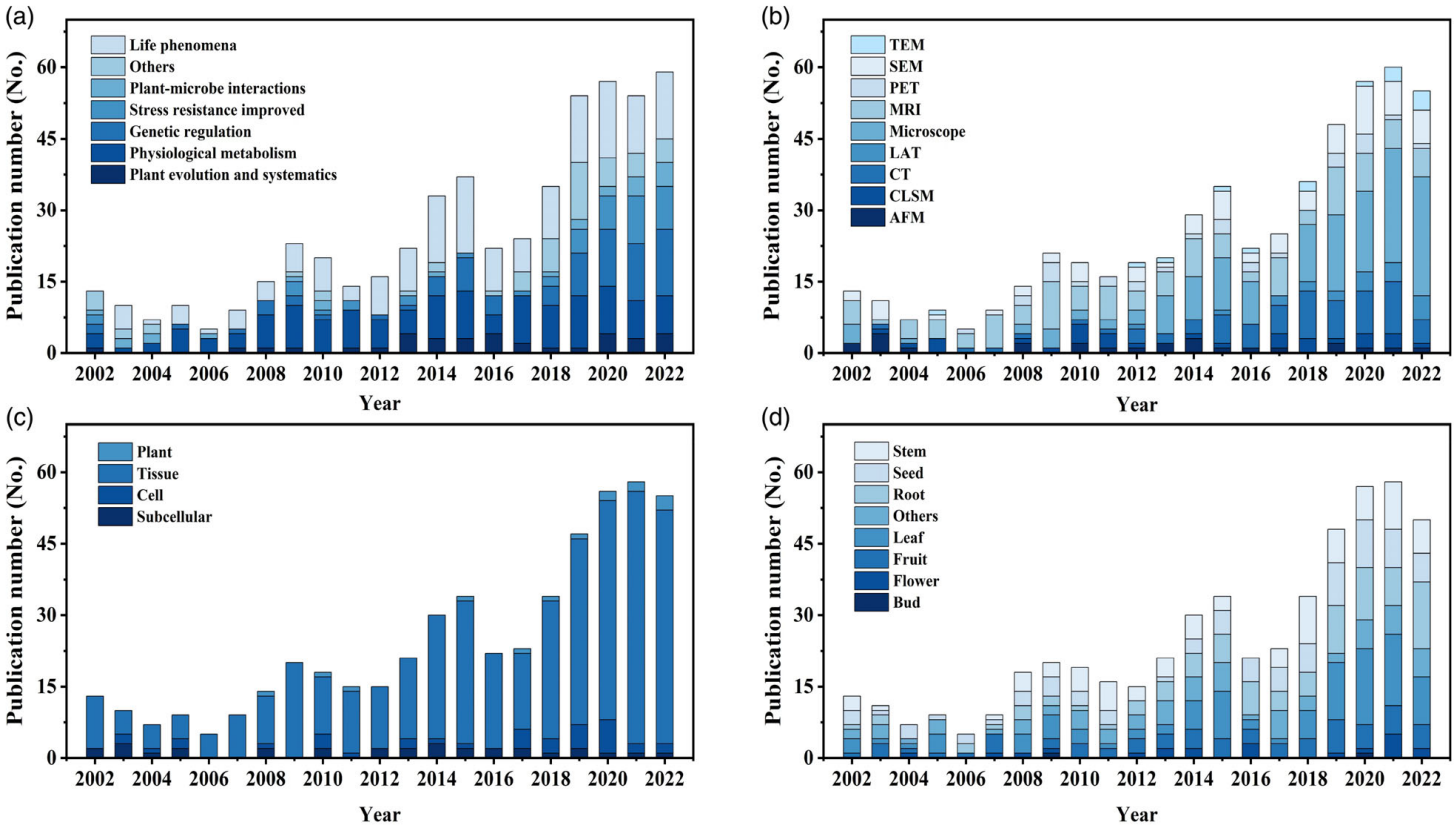
The statistics and categorization of published literature from 2002 to 2022 by different themes. (a) the number of yearly publications with different scientific questions; (b) yearly publications with different imaging modalities; (c) yearly publications with different research scales; (d) yearly publications with different plant organs. AFM, atomic force microscopy; CLSM, confocal laser scanning microscopy; CT, X‐ray computed tomography; LAT, laser ablation tomography; MRI, magnetic resonance imaging; PET, positron emission tomography; SEM, scanning electron microscopy; TEM, transmission electron microscopy.

The statistics from 2002 to 2022 reveal several trends in plant microphenotype‐related studies and publications. First, there has been a significant increase in the literature on plant microphenotypes over the past two decades, with the number of publications rising from 13 in 2002 to 55 in 2022 (Table S1). This growth can be attributed to the improvement of observation techniques, which diversified from 4 to 8 (Figure 2b). The growing interest in microphenotypes underscores their importance and potential for advancing knowledge in plant science, as they offer greater dimensionality, precision, and depth than macrophenotypes (Yu *et al*., 2020). Second, in terms of specific scientific issues, microphenotype research primarily focuses on revealing plant ‘life phenomena’. This type of research accounts for 30.6% of the literature, followed by studies on ‘physiological metabolism’ and ‘genetic regulation’, which account for 25.6% and 14.5% respectively. Notably, the application of microphenotypes in the study of ‘plant genetic regulation’ and ‘stress resistance improvement’ has increased in recent years. This is mainly due to the rapid development of modern biology, which has enabled in‐depth investigations at the level of genes and proteins. Microphenotypes have become an important component in the chain of evidence for studying specific biological processes (Figure 2a). However, it should be noted that the field of plant microphenotype research is still in its early stages, as evidenced by the prevalence of articles as the primary mode of disseminating findings (Table S1). Third, studies at the tissue, cell, and subcellular levels constitute 84.1%, 7.0%, and 6.2%, respectively, of all publications, with tissue‐level research still dominating (Figure 2c). Research on tissues in roots and leaves is more common, accounting for 16.8% of all publications, while research on flowers is less common, accounting for only 1.6% (Figure 2d). Fourth, optical microscopy currently plays a dominant role in the study of plant microphenotypes, but the use of other modalities, such as magnetic resonance imaging (MRI), computed tomography (CT), and scanning electron microscopy (SEM), has also grown in recent years (Figure 2b). Furthermore, scientists have realized that the combination of multiple‐scale phenotyping instruments can lead to a more precise and efficient unravelling of scientific inquiries (Table S1).

## Imaging modalities for plant microphenotypes

Compared to phenotyping technologies at the individual level or canopy level, the process of phenotyping at higher resolutions at the tissue and cellular scales is more complex (Hall *et al*., 2016). To overcome the limitations of ordinary optical microscopy, innovative imaging modalities ranging from the intercellular scale to the tissue and organ levels have been introduced (Feng *et al*., 2022). Techniques such as confocal laser scanning microscopy (CLSM), laser ablation tomography (LAT), high‐resolution X‐ray computed tomography (HRXCT) or micro‐CT (μCT), MRI, and positron emission tomography (PET) have been gradually applied to obtain high‐resolution images of organs, tissues, and cells in plants (Lv *et al*., 2022). Here, we summarize microimaging techniques that have been used for anatomical or functional phenotyping and highlight their capabilities for two‐dimensional (2D) or three‐dimensional (3D) imaging, destructive or noninvasive detection, and dynamic detection of developmental processes (Table 1).

**Table 1:** Summary of imaging modalities in plant science

Imaging techniques	Imaging medium	Range of scanning	Magnification	Phenotypic parameters	Advantages	Disadvantages	Reference
Atomic force microscope (AFM)	Laser beam/Electron beam	Micron level, ~100 μm^2^	Z direction: 0.1 nm; X, Y direction: 2 nm	Cellular geometry parameters of stomata or fibre	High‐resolution, 3D surface information	Greatly limited by sample factors, low scanning efficiency	Zhang, Calla, *et al*. (2021)
Scanning electron microscope (SEM)	Electron beam	Millimetre level, ~3 mm^2^	~1 nm	Geometry parameters of vessels, xylem parenchyma, fibres, cambium, stoma, etc.	High resolution, large depth of field	2D surface information, require a vacuum environment	Mullendore *et al*. (2010)
Three‐dimensional electron microscopy (3D‐EM)	Electron beam	Millimetre scale, tens of millimetres	~1 nm	Geometry and morphological parameters of cells and subcell of pollen grain, shoot, root, etc.	In situ microtomy Study cellular structures at nanometres resolution across millimetres of volume	Sample preparation complicated	Xu *et al*. (2017), Weiner *et al*. (2022)
Confocal laser scanning microscopy (CLSM)	Laser beam	Millimetre‐level, ~1 mm^2^	~100 nm	Cellular geometry parameters of xylem vessels, xylem parenchyma, xylem fibres, cambium, phloem fibres, etc.	High resolution, low noise, 3D information	Tedious sample preparation	Pound *et al*. (2012), Dhondt *et al*. (2013), Hall *et al*. (2016)
Laser ablation tomography (LAT)	Visible light	Centimetre level, ~0.1 mm × 1 cm	5, 10, 20×	Shape and geometry parameters of root cross‐section, cortex, and stele	Simple sample preparation, 3D information, high	Relatively low resolution	Burton *et al*. (2012), Strock *et al*. (2019), Strock *et al*. (2022)
Portable optical microscopes	Visible light	Centimetre level, ~0.01 mm × 1 cm	10, 50, 100, 200×	Shape, number and geometry parameters of stomata	Portable, low cost, simple operation	2D information, relatively low resolution	Liang *et al*. (2022)
Hyperspectral microscope imaging (HMI)	Visible light/laser beam	Centimetre level, ~0.01 mm × 1 cm	10, 50, 100, 200×	Chemical composition and contents parameters	Spectral information is combined with structural information	The accuracy of physiological index based on spectral data varies greatly	Fakhrullin *et al*. (2021)
X‐ray micro‐computed tomography (micro‐CT)	X‐ray	Centimetre level, ~30 cm × 50 cm	20 μm–1 μm	Shape and geometry parameters of seed, stem, leaf, root, vascular bundle, etc.	High spatial resolution, 3D nondestructive testing, high scanning efficiency	Poor contrast in plant tissue, ionizing radiation	Tracy *et al*. (2010), Tracy *et al*. (2012), Hughes *et al*. (2017), Zhang *et al*. (2018), Hu *et al*. (2020), Du *et al*. (2022)
Magnetic resonance imaging (MRI)	Radiofrequency pulse	Decimetre level	50 μm	Tissue and organ shape and geometry parameters, physiological indexes	High spatial resolution, physiological and metabolic index detection	Restricted by contrast agents	Jahnke *et al*. (2009), Borisjuk *et al*. (2012), Pajor *et al*. (2013)
Positron emission tomography (PET)	γ photon	Decimetre level, height ~ 11 cm, diameter ~ 7 cm	1–5 mm	Material metabolism, distribution and transportation parameters	Quantitative molecular imaging, physiological and metabolic index detection	High cost, low spatial resolution, ionizing radiation	Jahnke *et al*. (2009), Pajor *et al*. (2013)

Confocal laser scanning microscopy has become a crucial tool for obtaining cellular‐ and subcellular‐scale image data, as noted in studies by Pound *et al*. (2012) and Hall *et al*. (2016). CLSM offers fast, sensitive, and highly resolved imaging capabilities and is aided by a wide range of fluorescent probes and dyes, allowing for the observation of various cellular structures, such as cell walls, organelles, and proteins (Cutler *et al*., 2000; Pawley, 2006). For example, confocal imaging of Arabidopsis shoot and root tips has shed new light on meristem development and cell division (Hall *et al*., 2016; Kierzkowski *et al*., 2013). Truernit *et al*. (2008) also used CLSM to image the mesophyll and vascular tissues of leaves, enabling the analysis of three‐dimensional domains of gene expression at single‐cell precision. However, it should be noted that confocal imaging of living plant tissue is only practicable for thin (50–100 μm) and semi‐transparent organs, thus limiting its scope of application (Dhondt *et al*., 2013; Haseloff, 2003).

The most traditional way to obtain 3D information of relatively large areas of cells and tissues by electron microscope (EM) is serial section transmission EM (ssTEM), that is, the sequential imaging of serial plastic sections that were cut from a fixed and resin‐embedded sample using an ultramicrotome. This is a challenging and time‐consuming approach that requires the training and skill to collect hundreds of sequential thin sections (40–60 nm thick) on grids for imaging. Three‐dimensional electron microscopy (3D‐EM) has attracted considerable attention because of its ability to increase automated image collections through large tissue volumes using serial block‐face scanning EM, and achieve near‐atomic resolution of macromolecular complexes using cryo‐electron tomography (cryo‐ET) and sub‐tomogram averaging (Weiner *et al*., 2022). The technique has its roots in early electron microscopy advancements in the 1930s and 1940s, with the development of transmission electron microscopy (TEM) and scanning electron microscopy (SEM). Significant advancements in 3D‐EM techniques, such as serial sectioning and focused ion beam scanning electron microscopy (FIB‐SEM), have allowed for the reconstruction of three‐dimensional structures at the nanoscale and micrometre scale. Previous studies have indicated that 3D‐EM is a method that provides rapid examination of the anatomy of biological samples at the millimetre scale. This technology can improve the speed of SEM image acquisition, generate a large number of data sets in a shorter time, and achieve multiscale imaging of large numbers of plant samples (Guérin *et al*., 2019; Shen *et al*., 2020).

Laser ablation tomography utilizes a pulsed ultraviolet laser to ablate plant tissues and capture the exposed surface information. The system boasts a high‐resolution capability of up to the micrometre level, with a scanning range of 0.1 mm–1 cm (Burton *et al*., 2012). LAT offers the capability of 3D visualization and quantification of root anatomy with approximately 25 root segments per hour, increasing both the sample throughput and spatial resolution compared to traditional microscopy techniques (Strock *et al*., 2019). This technology has been successfully applied to investigate root anatomy at both the 2D and 3D scales in maize, wheat, sorghum, and other cereal crops (Strock *et al*., 2022).

High‐resolution X‐ray computed tomography has been frequently used to nondestructively image the internal structures of organs in 3D space. It allows the production of highly accurate and detailed images, thus improving the precision and dimensionality of monitoring internal structures for various organs. For example, both the internal and external 3D morphological traits of roots (Tracy *et al*., 2010, 2012), inflorescences (Li *et al*., 2020), tillers (Yang *et al*., 2011), and grains (Du *et al*., 2022; Hu *et al*., 2020; Hughes *et al*., 2017) have been measured from reconstructed data. To enhance the contrast of CT images caused by low attenuation values of fresh organs and tissue, a preprocessing protocol including fixation, dehydration, and staining has been introduced to achieve cellular‐resolution imaging in the laboratory (Mahesh, 2002; Pajor *et al*., 2013). Dhondt *et al*. (2010) recorded Arabidopsis hypocotyls at a spatial resolution of 0.85 μm, and high‐resolution CT images of maize roots, stems, and leaves can reach 1 μm (Zhang *et al*., 2018).

Magnetic resonance imaging uses the principle of nuclear magnetic resonance (NMR) to produce high‐resolution images of water protons and provide nondestructive structural information (Jahnke *et al*., 2009; Pajor *et al*., 2013). Given the high spatial resolution (up to 30 μm^3^ per voxel) and advantages in detecting the water content and transport in plants, MRI has been employed to investigate seed oil or water contents, internal structures of root or shoot systems, and the distribution of root water uptake and movement (Borisjuk *et al*., 2012). PET, on the other hand, uses short‐lived radioactive tracers, such as carbon isotopes, to measure and visualize metabolites (Garbout *et al*., 2011). As a complementary method to MRI, PET is a very powerful technology for detecting positron‐emitting radionuclides such as ^11^C, ^15^O, and ^13^N in plants in either 2D or 3D space (Jahnke *et al*., 2009). Although PET is capable of imaging the transport and distribution of photoassimilates in plants, it currently has a lower resolution, ranging from 1 to 5 mm (Pajor *et al*., 2013). MRI and PET have shown to be more suited for studying physiological processes such as water content/transport (MRI) and carbon assimilation/transport (PET) in plant tissues. In addition, MRI and PET can be used in conjunction with the structural data of plant tissues by the micro‐CT technique to provide new insights into the structural and functional traits of intact plants (Dhondt *et al*., 2010; Garbout *et al*., 2011).

Hyperspectral microscope imaging (HMI), infrared‐based microscope imaging (IFMI), and Raman microscope imaging (RMI) combine optical microscopy and spectral technology to nondestructively examine morpho‐chemical properties from single cells to intact tissues. Based on multiband spectral data, hyperspectral microscopy can realize the distribution and quantitative analysis of proteins and small molecules (Fakhrullin *et al*., 2021). Infrared‐based microscopy allows for the quantitative visualization of sucrose in individual vascular bundles and within a complex organ such as the stem, leaf, or seed (Hsiao and Huang, 2022). Raman microscopy has been employed to visualize cellulose and pectin in plant cell walls (Cui *et al*., 2023). In addition, confocal RM monitors specific chemical ‘fingerprints’ of the components of the cell membrane and organelles such as the nucleus (Nair *et al*., 2021). The integration and application of optical microscopes for the detection of plant microscopic traits have attracted increasing attention in recent years due to their advantages in cost‐effectiveness, functionality, and efficiency. Portable optical microscopes, which are low‐cost and compact, are ideal for imaging superficial structures such as root hairs, stomata, and trichomes, especially in the field where access to larger benchtop microscopes is limited (Liang *et al*., 2022). With further advancements in cost, compactness, and performance, portable optical microscopes will become a powerful tool for high‐throughput imaging across multiple disciplines, including ecology, physiology, and crop breeding (Strock *et al*., 2022).

## Artificial intelligence‐based analysis opens new avenues for microscopic phenotyping

In recent years, the resolution and speed of acquiring microphenotype information have undergone remarkable improvements due to the advancements and applications of optical, electronic, and chemical imaging technologies. An efficient approach for processing, analysing, and understanding massive microscopic imaging data has become increasingly important to catalyse biological discoveries (Conrad *et al*., 2021; Li *et al*., 2023; Pratapa *et al*., 2021). Fortunately, with the advancement of computer image analysis technology, scientists can now use computer programs to analyse these images and ‘better see what needs to be seen’ (Danuser, 2011). The rapid acceleration of machine learning, deep learning, and other artificial intelligence (AI)‐based computational tools has opened new possibilities for high‐throughput microscopic phenotyping applications (Pratapa *et al*., 2021).

Multimodal imaging techniques, such as optical, CT, MRI, and others, have revolutionized the microscopic imaging of plant tissues, cells, and their surrounding structures, with resolutions reaching submicron levels. Each imaging modality typically has its own image acquisition and analysis software that provides basic and general image processing and analysis functions. However, built‐in image analysis software often requires human intervention to extract useful information, and its automatic analysis capability is limited, thereby making the high‐throughput analysis of microscopic phenotypes challenging. To address this issue, open‐source image processing packages such as ImageJ (Schindelin *et al*., 2012; Schroeder *et al*., 2021) offer a range of image algorithms and extendable plugins, enhancing the flexibility of various microscopic imaging applications and becoming a popular tool in the scientific community. Additionally, specialized tools such as Huygens for fluorescence microscope images (Gambarotto *et al*., 2019; Göbel *et al*., 2020; Model *et al*., 2011) and Simpleware for X‐ray microcomputed tomography images (Rodrigues *et al*., 2009; Wang *et al*., 2012; Yuan *et al*., 2021) provide targeted and purposeful analysis for different types of microscopy images. However, due to the variations in the morphology, structure, and composition of organs and tissues at different stages and in different species, it is often challenging to obtain valuable information directly from these tools. Challenges include high learning costs, the need for active algorithm parameter adjustments based on user experience, and tedious human–computer interaction. Thus, the development of high‐throughput and intelligent phenotypic analysis pipelines and methods that cater to specific research objectives and problems is crucial for accelerating the automatic analysis and utilization of microscopy images.

In the field of microscopy image analysis, traditional image processing and classical machine learning techniques have been widely employed to process and analyse images with varying signal‐to‐noise ratios and resolutions. Applications include quantifying tissue and cell numbers and hypocotyl sizes (Campbell *et al*., 2017; Hall *et al*., 2016), stomata and pavement cell quantification of leaves (Jayakody *et al*., 2017; Möller *et al*., 2017), root visualization and quantification (Yamaura *et al*., 2022), and woody species classification (Rosa da Silva *et al*., 2017, 2022). Over the past decade, deep learning has emerged as a rapidly growing technology in plant microscopic imaging. From object detection and image segmentation to phenotypic trait analysis, deep learning techniques have been applied to solve problems that traditional image processing and machine learning algorithms struggle with. Convolutional neural networks (CNNs) are particularly powerful and can automatically train feature detectors and classifiers. Therefore, researchers are actively integrating adaptive machine learning and deep learning algorithms to create automated, high‐throughput phenotypic pipelines to enhance the robustness, accuracy, and automation of microscopic phenotypic analysis.

These methods often employ supervised learning techniques to directly extract high‐level semantic information from microscopy images, which requires the use of historical image data to construct feature sets or annotated data sets. Then, appropriate machine learning and deep learning models are selected for training. In recent years, numerous efficient phenotypic techniques have been developed for specific objects in microscopy images, such as leaf stomata (Liang *et al*., 2022; Toda *et al*., 2018), stem microfibril (Yusuke and Junji, 2021), vascular bundles (Du *et al*., 2016), and root (Jiang *et al*., 2022). Significant progress and improvement have been made in the technology and methods for phenotyping specific plant organs. Taking the analysis of maize stem vascular bundles as an example, the data acquisition method has gradually shifted from traditional paraffin section imaging to industrial CT scanning imaging (Zhao *et al*., 2018), which has greatly improved the throughput and standardization of data acquisition. The vascular bundle detection method has also shifted from relying on image analysis of vascular bundle features to data‐driven object detection network models (Du *et al*., 2021), which has greatly enhanced the robustness and ease of use of phenotyping technology. In addition, deep learning models with powerful semantic analysis capabilities, such as the Segment Anything Model (SAM) (Kirillov *et al*., 2023), are being introduced into microscopic phenotyping, which will further improve the automatic extraction and understanding of rich semantic content. These applications highlight the enormous potential of using deep learning and microscopy to capture and quantify meaningful traits at the tissue and cellular scale in large‐scale data analysis tasks (Table 2).

**Table 2:** Summary of microscopic phenotyping extraction based on machine learning and deep learning of plants

Method type	Algorithm and models	Microscopic images	Traits	Species	Organ	Reference
Machine learning	Adaptive thresholding	CLSM images	Spatial, intensity and Haralick of cell walls and spindle‐shaped cellular organelles	Arabidopsis	Cotyledons	Basak *et al*. (2021)
	Cascade object detection (COD) algorithm	Optical microscope images	Pore area, eccentricity	Grape	Leaf	Jayakody *et al*. (2017)
	/	Optical microscope images	Cell size, shape, and number of xylem tissue	Poplar	Hypocotyl	Campbell *et al*. (2017)
	Random forest classification	CLSM images	Anatomical traits of xylem vessels, xylem parenchyma, xylem fibres, cambium, phloem fibres, phloem parenchyma, and cortex	Arabidopsis	Hypocotyl	Hall *et al*. (2016)
	Canny edge detector	X‐ray μCT images	Visualize and quantitatively analyse the rhizoid system	Physcomitrella	Root	Yamaura *et al*. (2022)
	Global thresholds	CLSM images	Twenty‐seven shape features of pavement cells	Arabidopsis	Leaf	Möller *et al*. (2017)
	SIFT algorithm and connected‐component labelling	Optical microscope images	Pore size distributions	Eighteen species from the Fagaceae	Stem	Kobayashi *et al*. (2019)
	Support vector machines (SVMs)/Local phase quantization (LPQ)/Multi‐view random forest model	Optical microscope images	Automate wood species identification	Commercial wood species	Stem	Martins *et al*. (2013), Rosa da Silva *et al*. (2017, 2022)
Deep learning	FPN + Faster R‐CNN/Faster R‐CNN/Mask R‐CNN	Optical microscope images	Pore number, density, length, width, area, and pore eccentricity	Maize, black poplar, dayflower, etc.	Leaf	Li *et al*. (2019), Song *et al*. (2020), Jayakody *et al*. (2021), Liang *et al*. (2022)
	R‐CNN + U‐Net/R‐CNN	Optical microscope images	Stomata number, stomatal complex area, epidermal cell number, etc.	Wheat, maize, and sorghum	Leaf	Zhu *et al*. (2021), Xie *et al*. (2021), Bheemanahalli *et al*. (2021)
	F‐CNN	SEM images	Stomata number, width, and length	Rice	Leaf	Bhugra *et al*. (2018a,b)
	D‐CNN	Optical microscope images/Bright field/SEM images	Stomata number	Maize, poplar, ginkgo, etc.	Leaf	Aono *et al*. (2021), Fetter *et al*. (2019)
	CNN	Optical microscope images/SEM images	Stomata classification, aperture	Dayflower, ginger, etc.	Leaf	Toda *et al*. (2018), Andayani *et al*. (2020), Millstead *et al*. (2020), Li *et al*. (2022)
	Single shot multibox detector (SSD)/Cascade classifier	Optical microscope images	Stomatal density	Soybeans, oak	Leaf	Vialet‐Chabrand and Brendel (2014), Sakoda *et al*. (2019)
	CNN	X‐ray μCT images	3D architecture segmentation (leaf: epidermis, mesophyll, bundle sheath, and the air space)	Walnut	Leaf	Rippner *et al*. (2022)
	YOLO network	Optical microscope images	Stomatal number	Common bean, soybean, barley	Leaf	Casado‐García *et al*. (2020)
	VGG19	Digital microscope images	Stomatal density	Common tropical tree	Leaf	Meeus *et al*. (2020)
	Attention U‐Net and Inception	Optical microscope images	Number, density, length and width of pores	Wheat, poplar	Leaf	Gibbs *et al*. (2021)
	CNN	Handheld microscope images	Classifies images of cotton leaves according to hairiness	Cotton	Leaf hairiness	Rolland *et al*. (2022)
	SegNet	Micro‐CT‐RGB images	Culm morphological traits	Rice	Stem	Wu *et al*. (2021a)
	CNN	Polarized optical microscope images	Micro fibril angle (MFA)	Thujopsis spp.	Stem	Yusuke and Junji (2021)
	Generative adversarial network (GAN)	Optical microscope images	Shape and pore size	Eighteen species from the Fagaceae	Stem	Lopes *et al*. (2021)
	U‐Net	Micro‐CT images	Shape and geometry parameters of stem cross‐section, number, shape, geometry, and distribution parameters of vascular bundles	Maize	Stem	Du *et al*. (2022)
	CNN	CLSM images	Single‐cell fluorescence quantification	Rice	Root	Jiang *et al*. (2022)
	3D U‐Net	CLSM images	Volumetric segmentation of cell cycle markers and quantify cell division	Arabidopsis	Root	Khan *et al*. (2020)
	CNN	CLSM and light sheet microscopes	Number and volume of ovule primordia, lateral roots, and epidermal cell	Arabidopsis	Ovule, roots, and leaf	Li *et al*. (2022)
	Mask R‐CNN	X‐ray CT images	3D panicle and grains	Rice	Panicle	Kong and Chen (2021)
	RAUNet‐3D	Micro‐CT images	Shape, geometry, and structure parameters of kernel, embryo, endosperm, and cavity	Maize	Kernel	Du *et al*. (2022)
	CNN/YOLO	Optical microscope images	Classification of pollen grain	Alnus, Betula, and Corylus	Pollen	Kubera *et al*. (2021, 2022)
	CNN	High‐resolution digital microscope images	Storage time prediction based on the evolutionary characteristics of the rind oil glands	Orange	Fruit	Gao *et al*. (2022)
	Transfer learning and CNN	CLSM images	Identification and prediction of chloroplasts, mitochondria, or peroxisomes with morphological defects	Arabidopsis	Leaf	Li *et al*. (2021a)

## Applications of microphenotypes in biophysical and biochemical processes of water and carbon cycling

### Quantifying the structures and dynamics of plant vascular tissue and cells

In multicellular organisms, individual components are interconnected in complex biological networks that help these systems maintain homeostasis for development and environmental adaptation (Gosak *et al*., 2018). Phenotyping traits related to water and carbon fluxes in plants have evolved from studies focusing on macro traits at the organ, plant, and canopy levels to those focusing on microphenotypes below the suborgan level. The plant vasculature is the crucial intercellular structural network that functions in nutrient and water distribution and mechanical support. Quantitative information on the structure and multiscale network of cells and tissue is essential for understanding plant vascular system development at a range of temporal and spatial scales. For instance, by imaging with CLSM, SEM, and CT technology, vascular development patterning in the root, leaf, and stem in Arabidopsis has been established, and the genetic and hormonal networks that cooperate to orchestrate vascular development have been analysed (Bassel, 2019; Lucas *et al*., 2013). Moreover, analysing whole vessel networks will allow us to better understand how the distribution of intervessel connections influences hydraulic conductivity, the movement of pathogens and embolisms, and the ability to adapt or acclimate to a changing environment. In one example, using X‐ray computed tomography, vessel dimensions and the distribution of intervessel connections of the stem were reconstructed, and xylem embolism and water transport properties were quantified (Brodersen *et al*., 2011). In another example, a recent study quantified the stiffness of intervessel pit membranes by applying an AFM‐based indentation technique called ‘Quantitative Imaging’ and paired it with magnetic resonance imaging to visualize and quantify embolised vessels (Carmesin *et al*., 2023).

### Genetic analysis of important microscopic traits

**Figure 3:**
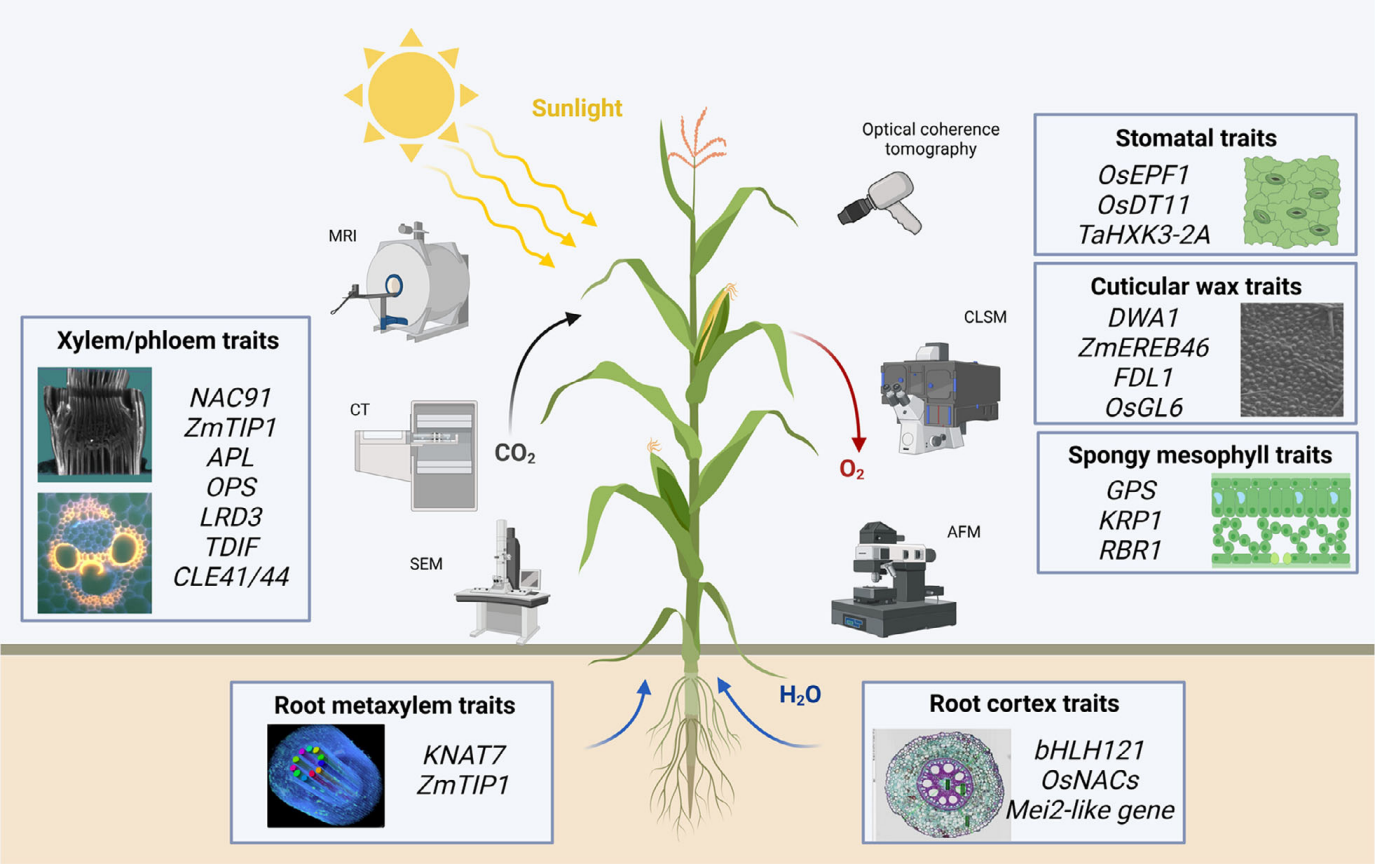
Characterization of traits involved in water and carbon transport processes within plants and some key genes that have been identified. AFM, atomic force microscopy; CLSM, confocal laser scanning microscopy; CT, X‐ray computed tomography; MRI, magnetic resonance imaging; SEM, scanning electron microscopy.

The measurement of each genotype through sequencing and downstream macro outputs through phenotyping enables links between the molecular and organismal scales (Atwell *et al*., 2010). However, the ability to map genotypes to phenotypes represents a grand challenge in biology (Bassel, 2019). For example, out of 40 000 functional genes in maize, fewer than 200 genes have been cloned and functionally verified (Wang and Cai, 2019). Our ability to understand the mechanistic basis by which genetic changes lead to phenotypic consequences remains limited. A quantitative view of organ architecture and the pursuit of investigation to understand the functional consequences of cellular configurations may contribute towards the bridging of genotype–phenotype mapping. Emerging microphenotyping technologies, such as portable microscopy, LAT, and CT, combined with multiomics studies help identify several genetic resources related to water and carbon transport processes in plants (Figure 3). The vascular system is the major organ responsible for water uptake and transportation in plants. With the precise identification of microphenotypic traits, an increasing number of genes that regulate microphenotypes of vasculature, such as the metaxylem vessel number (*KNAT7*, *ZmTIP1*), stele area (*OsNACs*, Mei2‐like gene), cortical cell (*bHLH121*), and stem xylem and phloem traits (*NAC91, ZmTIP1, CLE41/44*, etc.), are being uncovered, which provides insights for the genetic improvement of crop yield and stress resistance (Barrieu *et al*., 1998; Li *et al*., 2021b; Liu *et al*., 2022; Schneider *et al*., 2023).

Photosynthesis is another attractive target for improving crop yields. Maintaining high rates of photosynthesis in leaves requires the efficient movement of CO_2_ from the atmosphere to the mesophyll cells inside the leaf where CO_2_ is converted into sugar (Théroux‐Rancourt *et al*., 2021). Therefore, testing how the structure influences leaf function requires the detailed characterization of the morphological and anatomical structure of the leaf tissue. Borsuk *et al*. (2022) analysed the 3D cellular geometry of spongy mesophyll in 40 species belonging to 30 genera using X‐ray microcomputed tomography imaging and found that the structural organization of spongy mesophyll is a key trait influencing leaf economy. Genome size determines the sizes of stomatal guard cells and mesophyll cells, collectively allowing for higher photosynthetic capacity by optimizing the hydraulic and diffusive pathways in the leaf (Simonin and Roddy, 2018; Théroux‐Rancourt *et al*., 2021). With the development of methods for obtaining microphenotypes of mesophyll cells, typical and representative genes regulating spongy mesophyll (GPS, KRPA, and RBR1) have been identified (Ren *et al*., 2019).

### Functional–structural plant modelling by integrating microphenotypes

Functional traits are crucial in understanding the mechanisms underlying fitness and evolution from an ecological perspective and in increasing plant productivity from an agricultural perspective. Despite the remarkable progress in microphenotyping, most of these studies focused on structural traits with limited and qualitative expression of their function, resulting in a knowledge gap between microphenotypes and macrophenotypes for complex traits. Functional–structural plant models (FSPMs), explicitly describing the development over time of the 3D architecture or structure of plants as governed by physiological processes that, in turn, depend on environmental factors, have become a powerful tool in plant science and evolved to the multiscale in the past 30 years (Soualiou *et al*., 2021). With the continuous development of depth in phenotypic information acquisition, FSPMs can be downscaled to the tissue, cellular, and molecular levels or upscaled to the whole plant and ecological levels, thereby allowing the breakdown of complex macrotraits into specific microtraits and translating microtrait contributions to macrotraits. FSPMs showed great potential in accelerating precision breeding and basic plant science by (i) providing crop ideotypes efficiently for optimizing the resource distribution, (ii) guiding molecular design breeding by linking a molecular basis to plant phenotypes, and (iii) interacting with three‐dimensional (3D) architectural traits to interpret the outcome of complex processes and guide the measurement and modification of targeted traits.

#### Carbon

To better understand carbon dynamics in plants and evaluate the effect of specific microphenotypes on source capacity, transport resistance, and sink strength, mechanistic models have been created. For example, a 3D model incorporating leaf geometry, light propagation, CO_2_ diffusion, and photosynthesis was developed for tomato leaves to analyse the impact of photosynthetic capacity on CO_2_ fixation (Ho *et al*., 2016). By refining the 3D model of leaf anatomical structure (e.g. vascular bundles) using transmission electron microscopy and X‐ray micro‐CT, the eLeaf was developed to quantify the influence of morphological traits on leaf photosynthesis (Xiao *et al*., 2023). Based on the transport‐resistance models that are widely considered suitable to explain the transport of carbohydrate from sources to sinks (Thornley, 1991), Allen *et al*. (2005) developed an FSP model L‐PEACH to simulate the response of a tree to fruit thinning and water stress condition and Gu *et al*. (2018) developed the CottonXL to simulate the heterogeneity of carbohydrate as an emergent property with optimizing the transport resistance coefficient mathematically. Instead of simplifying or optimizing the resistance or conductance, Knoblauch *et al*. (2016) calculated the sieve tube specific conductivity at different position of stem by the Hagen–Poiseuille equation after measuring phloem area, transport distance, and sap flow velocity of labelled C, providing support for parameterizing transport resistance model (Minchin and Lacointe, 2005). The integration of microphenotypes into FSPMs will be valuable for improving the accuracy of predictive phenotyping for complex macrophenotype such as RUE and the understanding of the underlying mechanism of carbon dynamics.

#### Water

The modelling of water fluxes in the soil–plant‐atmosphere system is crucial in comprehending the impact of environmental variables and plant hydraulic traits on plant growth and the water cycle under global change (Lynch *et al*., 2014; Mencuccini *et al*., 2019). Integration of microphenotyping into hydraulic modelling offers advanced and powerful multiscale models to unravel micro traits that regulate the emergent macrophenotypes such as transpiration. Fox example, the integration of hydrology module into the FSP model for root OPENSIMROOT can provide 3D distribution of water uptake by root segments (Postma *et al*., 2017), and this model has been later applied to evaluate the effect of root architecture on the depth of root system under drought stress (Strock *et al*., 2021). As another example, Schnepf *et al*. (2018) developed a root architecture model CRootBox and coupled it to a model of soil water movement and a model of water flow in plant, in which the water uptake was calculated based on root architecture, xylem pressure, and soil saturation. By taking the 3D structure of xylem and connectivity of xylem network into account, Loepfe *et al*. (2007) developed a xylem hydraulic model that is capable of simulating water transport in the vascular system of a plant and used the model to explore the effect of connectivity on hydraulic conductivity and the vulnerability to embolism. Furthermore, the integration of physiological traits into modelling allows investigating the effects of ABA synthesis and circadian oscillation of root hydraulic conductance on water uptake (Tardieu *et al*., 2015) and even on fruit growth and quality by coupling with carbon model (Zhu *et al*., 2019) (Table 3).

**Table 3:** Summary of mechanistic models of water and carbon dynamics in plants

Models	Processes	Platform/Programming language	Applications	Scale	Species	Reference
Stochastic, Markov chain and mechanistic modelling	Lateral root initiation and development	OpenAlea	Testing possible hypothesis underlying root branching	Tissue and organ	Arabidopsis	Lucas *et al*. (2008)
HYDRUS and RSWMS	Soil water absorption	OpenSimRoot	Simulating 3D water uptake profile	Organ and tissue	Maize	Postma *et al*. (2017)
Richard's equation and Doussan model	Root water uptake	CRootBox	Simulating water flow in soil, water uptake by roots, and water flow in roots	Organ and tissue	Sorghum	Schnepf *et al*. (2018)
3D conduit and darcy's law	Water transport in xylem	Java	Evaluate the importance of xylem network structure in water transport	Tissue	Acer negundo	Loepfe *et al*. (2007)
Tardieu–Davies model	Stomatal aperture, transpiration, and hydraulic conductance	Rstudio	Simulating variables of hydraulic processes with genotype‐specific parameters	Organ and tissue	Maize	Tardieu *et al*. (2015)
eLeaf	Light propagation, internal CO_2_ distribution, and net photosynthesis rate	COMSOL Multiphysics and MATLAB	Quantifying effects of morphological and biochemical properties on leaf photosynthesis	Cell	Rice	Xiao *et al*. (2023)
3D microscale model of leaf photosynthesis	Light propagation, CO_2_ transport within leaf and net photosynthesis rate	MATLAB	Investigating the influence of photosynthetic capacity within leaf on photosynthesis rate in relation to CO2 levels	Cell and tissue	Tomato	Ho *et al*. (2016)
Transport‐resistance model	Carbon transport and partitioning to organs	–	Reproducing the detailed dynamics of carbon transport and partitioning hierarchy as an emergent property	Tissue and organ	Woody plants and barley	Minchin and Lacointe (2005)
Hydraulic resistor model	Phloem translocation	–	Quantifying the effect of sieve plates on the hydraulic resistance of phloem	Tissue	Nineteen plant species	Jensen *et al*. (2012)
L‐Peach	Production, storage, and mobilization of carbohydrates.	L‐studio	Investigating the response to fruit thinning and water condition	Organ and plant	Peach	Allen *et al*. (2005)
CottonXL	Production, transport, storage, and allocation of carbohydrates.	GroIMP	Reproducing the spatial heterogeneity in carbohydrate availability in cotton	Organ and plant	Cotton	Gu *et al*. (2018)
GrapevineXL	Fruit growth and sugar accumulation in grape	GroIMP	Evaluating the contribution of different physiological processes and environmental variables to berry growth	Tissue, organ, and plant	Grape	Zhu *et al*. (2019)

## Challenges and future perspectives

**Figure 4:**
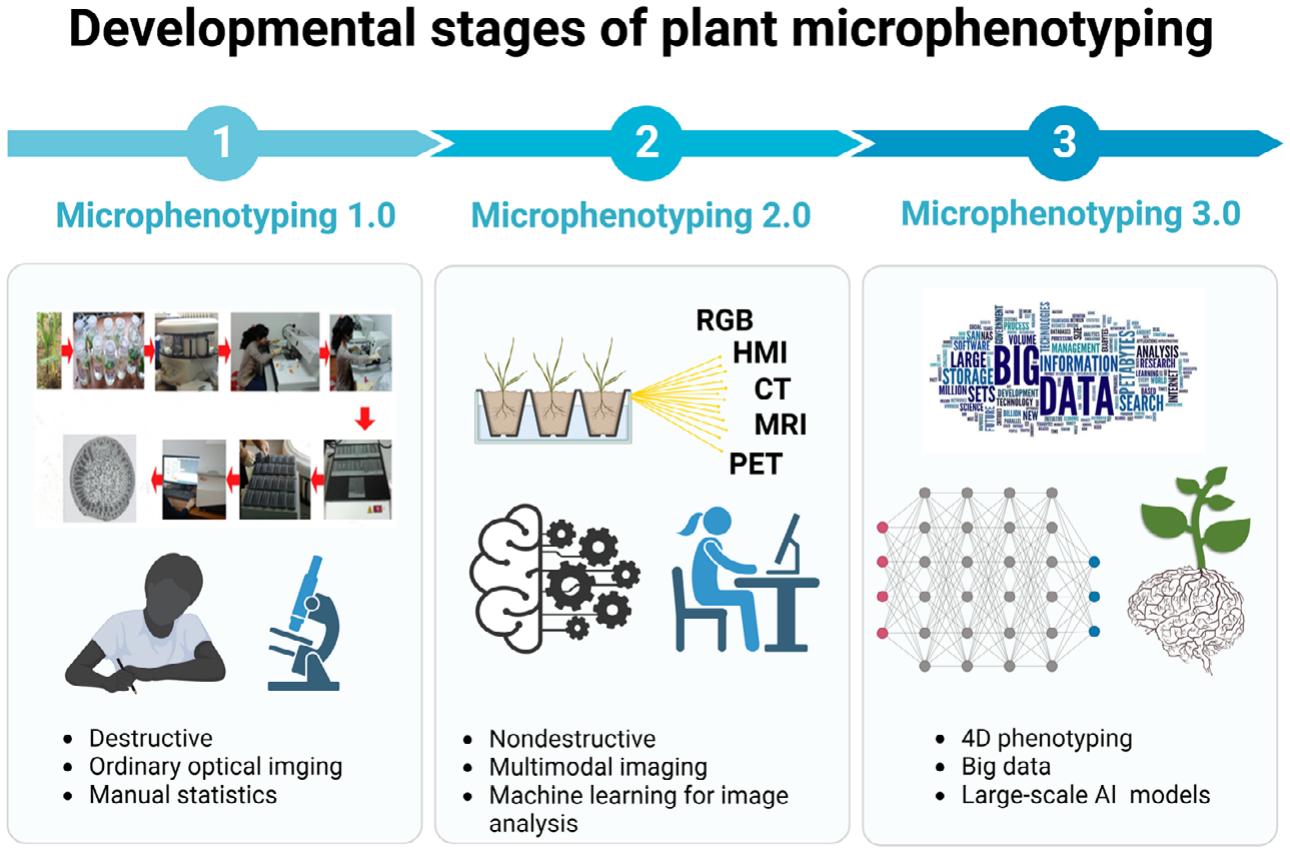
Three developmental stages of plant microphenotyping. Plant microphenotype efforts can be divided into three existing or near‐future stages based on the techniques involved. Microphenotyping 1.0: invasive sampling methods such as sectioning, conventional optical imaging, and manual analysis (statistics). Microphenotyping 2.0: nondestructive testing, multimodal imaging (RGB/CT/MRI, etc.), and machine learning for image analysis (semi‐automated/automated). Microphenotyping 3.0: in situ temporal phenotyping (4D), multimodal big data (images, videos, text, etc.), and AI big models. CT, computed tomography; HMI, hyperspectral microscope imaging; MRI, magnetic resonance imaging; PET, positron emission tomography; RGB, red–green–blue.

According to our analysis of the available technologies and methodologies, the development of plant microphenotyping can be categorized into three developmental stages: Microphenotyping 1.0, 2.0, and 3.0 (Figure 4). Microphenotyping 1.0 relies predominantly on classical histological sectioning and optical microscopy, which are constrained by destructive sampling and limited efficiency. In contrast, Microphenotyping 2.0 has witnessed significant advances enabled by the advent of novel nondestructive imaging modalities and semi‐automated/automated computational analysis. These innovations promise great potential for elucidating previously intractable relationships between microscopic phenotypes, molecular mechanisms, and macroscopic traits. In particular, microphenotyping has played an important role in constructing detailed quantitative models of gene–phenotype–environment (G × E × M) interactions and enhancing predictive crop models by synthesizing multivariable determinants underlying microphenotypes. With the rapid progress of modern artificial intelligence, big data, and associated technologies, Microphenotyping stage 3.0, characterized by AI‐empowered microscopic 3D/4D phenotyping quantification and prediction, may soon arrive. In summary, our analysis draws a profile of the evolution of microscopic phenotyping defined by available techniques and driven by technological innovation that aims to build large‐scale, multidimensional plant microphenotypes to decode G × E × M interactions. To achieve this ambitious vision, the following sections discuss the challenges that need to be addressed and summarize potential future trends.

### Microscopy image analysis based on AI technologies

In recent years, the multimodal image data (RGB, CT, MRI, HMI, etc.) collected in plant microscopic phenotyping studies have grown exponentially both in quality and quantity. Meanwhile, tremendous progress has also been made in microscopy image analysis techniques. However, analysing such complex and enormous microscopy image data sets is laborious and time‐consuming, and analysis results tend to have considerable intra‐ and interobserver variabilities. Efficiently analysing images, accurately extracting valuable microscopic phenotypes, and quantitatively characterizing the abundant microscopic structures with cellular/tissue resolution contained in the images remain enduring challenges for researchers.

Currently, the integration of deep learning techniques and microscopy image analysis has brought tremendous changes and opportunities for plant microscopic phenotyping analysis. Deep learning techniques represented by CNNs, with their advantages in automatic feature learning, high accuracy, and generalization capabilities, enable more precise and efficient microscopy image analysis. However, developing high‐performance deep learning models usually requires large‐scale manually annotated training data and intensive computational resources. To enable the constructed deep learning models to better fit microscopic phenotyping tasks, exploiting unsupervised learning and transfer learning techniques to mitigate laborious data annotation and achieve automatic feature representation learning from unlabelled data is an evident demand. In addition, reinforcement learning methods can be seamlessly integrated with microscopy image analysis by dynamically adjusting and optimizing models according to phenotyping tasks, improving their performance and robustness. With the substantial expansion of microscopy image data and model parameters, pretrained foundation models in a self‐supervised or semi‐supervised manner can greatly facilitate the practical applications of various microscopic phenotyping analysis tasks.

### Integration of multimodal imaging techniques

Plant phenotypic observation at the cellular and tissue levels has progressed from destructive sectioning to noninvasive detection, from single optical to multimodal imaging, and from low‐throughput single‐sample analysis to high‐throughput automated workflows. Systematic, heterogeneous, dynamic multiscale and multi‐dimensional information is instrumental in overcoming limitations posed by different scales in scientific research. In this context, multi‐factor correlation, the integration of multiple imaging modalities and approaches, is expected to emerge as a prominent and indispensable research methodology. Such as for tracking ROI traits, traditional methods (light microscopy) prefer to search for a needle in a haystack, relying on laborious and experienced judgement that results in low efficiency and poor accuracy. HRXCT was found to reveal impressive detail in tissue, allowing for the identification of tissue landmarks that can be subsequently used to guide data collection in the SEM (Bushong *et al*., 2015; Lu *et al*., 2021). This protocol enables fast large‐scale, nanometre‐resolution three‐dimensional imaging of subcellular structures in a targeted large‐volume tissue. In addition, RGB, MRI, and PET can be used in conjunction with the structural data of plant tissues by the HRXCT technique to provide new insights into the structural and functional traits of intact plants (Dhondt *et al*., 2010; Garbout *et al*., 2011; Wu *et al*., 2021a).

### Mining time‐series 3D phenotypes of tissues and cells

State‐of‐the‐art imaging instruments and image analysis tools in microscopy have streamlined and refined the acquisition of 3D phenotypes in a direct way instead of manually extracting phenotypic traits and arbitrarily mapping 3D geometry and tissue properties from 2D micro images. A previous study indicated that the estimated mesophyll surface area exposed to airspace in the 2D approach can be 15%–30% lower than that in the 3D approach (Théroux‐Rancourt *et al*., 2017). However, a phenotype is the product of dynamic interactions between the genotype, environment, and management in a 3D space. The challenge lies in the time series of high‐resolution and spatially explicit phenotyping, which is termed 4D phenotyping. For example, emerging technologies such as multiangle image acquisition, three‐dimensional reconstruction and cell segmentation‐automated lineage tracking (MARS‐ALT), and digital adaptive optics scanning light field microscopy (DAOSLM) (Fernandez *et al*., 2010; Wu *et al*., 2021b). This technique allows for phenotyping traits at a given stage and quantitatively evaluating morphogenesis at tissue and cell resolution during development (Fernandez *et al*., 2010). Another advantage is to improve the interpretability and reliability in mapping interactions between genotypes, environments, and phenotypes by coupling with process‐based modelling (Li *et al*., 2023). Hence, the time‐series high‐resolution phenotyping of cells and tissues will not only add temporal dimensions to the phenome at the microscale but will also complement multiscale computational models.

### Bridging the gap between micro‐ and macrophenotypes

Despite great advances in high‐throughput acquisition and the intelligent extraction of both micro‐ and macrophenotypes, one of the key challenges remains in narrowing the knowledge gap between micro‐ and macrophenotypes. For instance, the sieve tube conductivity of phloem flow systems in different species and their response to environmental changes have been quantitatively determined by integrating SEM techniques with the mechanistic modelling of phloem translocation, but their effects on favourable macro traits such as carbohydrate allocation remain unknown and need to be investigated (Mullendore *et al*., 2010). Multiscale computational models coupled with increasingly accurate structural data sets has been proposed to be a promising approach in understanding drug action through the hierarchy of biological complexity (Amaro and Mulholland, 2018), and this may enlighten us in tackling the microscale heterogeneity across spatiotemporal scales. In a pioneering work in plant science, a multiscale model of Arabidopsis integrating processes including genetic regulation, carbon metabolism, organ development, and plant growth was developed and capable of accurately predicting plant biomass of different Arabidopsis accessions (Chew *et al*., 2014). In one example that further integrating with microphenotype, Sidhu *et al*. (2023) developed a functional–structural modelling platform for root anatomy, and integrated this model to a plant level model to evaluate the role of anatomical phenes in improving the overall performance of root system under low nitrogen availability. Another case on leaf is that a 3D reaction–diffusion model for leaf photosynthesis by integrating anatomical and physiological phenotypes was developed and used to evaluate the influence of leaf anatomical and biochemical traits on leaf‐level photosynthesis (Xiao *et al*., 2023). The combination of multiscale modelling and microphenotype is expected to overcome the hierarchical complexity when elucidating the mechanisms of emergent phenomena in the fundamental plant science.

### Accurate and quantitative analysis of genetic effects based on microphenotypes

In recent years, multiomics joint analysis has become a crucial tool for uncovering the genetic regulatory mechanisms of specific biological phenomena. For example, metabolomics‐based genome‐wide association studies (mGWAS) and joint analysis of transcriptomes and genomes have propelled development in the field of life sciences, allowing the identification of many trait functional genes, such as those regulating the height of corn (Le *et al*., 2022; Wang *et al*., 2023). However, the knowledge network between genomic information and macrophenotypes cannot form a complete evidence chain. The study of microphenotypes can help us delve deeper into gene functions, enabling us to link genes and phenotypes more accurately, thoroughly analyse gene functions, and clarify how gene mutations influence specific life processes. For example, the elongation pattern of stalks arises from the variable growth of individual internodes driven by cell division, cell expansion, and vascular bundle formation comprising the maize stalk. Through constructing transcriptional regulatory networks with a fine spatiotemporal resolution of different functional tissue of stems, the key modules and candidate genes involved in cell and vascular bundle elongation and division that determine stalk length and thickness in maize were uncovered (Le *et al*., 2022). Perfecting the genetic regulatory network of specific traits through the study of microphenotypes will be the development trend of this field. Emerging single‐cell sequencing technologies applied to tissues offer a unique opportunity to study the relationships between microphenotypes, gene/protein expression levels, and spatial distribution. The correlation patterns between morphology and gene expression can be extracted and quantified as spatial transcriptomics data are introduced, significantly improving accuracy (Pratapa *et al*., 2021). In addition, the integrative analysis of big data of macro and microphenomics and system biology models may facilitate the precise appraisal of complex traits such as light, water, and fertilizer use efficiency and the systematic understanding of G × E × M interactions.

### Standardization, reuse, and sharing of microphenotypic data

The advancements in phenotyping technology in the field of plant sciences have greatly expanded the volume and depth of data generated over the last few decades (Arend *et al*., 2022). There is a growing focus on sharing data in a manner that is findable, accessible, interoperable, and reusable (FAIR) (Wilkinson *et al*., 2016). To facilitate this, the Plant Genomics and Phenomics Research Data Repository (e!DAL‐PGP) was launched in 2016 and has already published 250 comprehensive and diverse plant data sets (Wilkinson *et al*., 2020). PHIS, established by INRA (the French National Institute for Agricultural Research), stores highly heterogeneous (e.g. images, spectra, and growth curves) and multispatial and temporal‐scale data (leaf to canopy level) originating from multiple environments (field, greenhouse) (http://www.phis.inra.fr/). ZEAMAP, a database including 21 agronomic traits, 31 kernel lipid content‐related traits, 19 kernel amino acid content‐related traits, and 184 known metabolites of maize kernels from the AMP (an association mapping panel), was established by Huazhong Agricultural University in China (Gui *et al*., 2020; Yang *et al*., 2011). Although these data sets have greatly enhanced the sharing of phenotypic information, there is still a lack of data at the micro level. Since images from different imaging modalities have great data variability between different model organisms, experimental conditions, and laboratories, it is important to establish common rules of normalization and interpretation for phenotypic data obtained by different microscopy techniques. These validation data sets must be representative enough and cover a wide enough range of samples and imaging conditions (Li *et al*., 2023). Moreover, to facilitate the sharing of large‐scale microphenotypic data, web platforms supporting the online preview and downloading of high‐dimensional imaging data and high‐efficiency storage data formats are needed. For instance, the International Society for Advancement of Cytometry (ISAC) established the Flow Cytometry Standard (FCS) for describing flow cytometry data. This standard defines the format, metadata, and storage of flow cytometry data files to ensure data interoperability among different flow cytometry devices and software. In another case, CREAF initiated the SAPFLUXNET project, which contains a global database of sap flow and environmental data compiled from contributions from researchers worldwide (Poyatos *et al*., 2016). It is no doubt that, with more ‘FAIR’ microphenotypic data and AI models, microphenotyping has stepped into the era of Microphenotyping 3.0 and will largely promote functional genomics and plant science.

## Conflict of interest

The authors have no conflict of interest to declare.

## Author contributions

Y Zhang, S Gu, and J Du drafted and revised the article. C Zhao, X Guo, and W Yang proposed the conceptualization of this review and revised the article. G Huang, J Shi, X Lu, and J Wang collected literature and analysed data. Y Zhang, S Gu, J Du, and G Huang made figures and tables. C Zhao, X Guo, and W Yang are in charge of supervision and funding acquisition. All authors read and approved the final article.

## Supporting information


**Table S1** Literature compilation on plant microphenotype.
